# Chemical Composition of the Essential Oil of Mastic Gum and their Antibacterial Activity Against Drug-Resistant *Helicobacter pylori*

**DOI:** 10.1007/s13659-014-0033-3

**Published:** 2014-07-19

**Authors:** Tomofumi Miyamoto, Tadayoshi Okimoto, Michihiko Kuwano

**Affiliations:** 1 Department of Natural Products Chemistry, Graduate School of Pharmaceutical Sciences, Kyushu University, Fukuoka, Japan; 2 Department of Gastroenterology, Oita University Faculty of Medicine, Oita, Japan; 3 Laboratory of Molecular Cancer Biology, Graduate School of Pharmaceutical Sciences, Kyushu University, Fukuoka, Japan

**Keywords:** Mastic gum, *Pistacia lentiscus*, Anti-*Helicobacter pylori*, *α*-terpineol, (*E*)-methyl isoeugenol

## Abstract

Mastic gum is derived from the tree named *Pistacia lentiscus* that is grown only in Island Hios of Greek. Since Mastic was first reported to kill *Helicobacter pylori* (*H. pylori*) in 1998, there has been no further study to elucidate which component of mastic specifically shows the antimicrobial activity against *H. pylori*. In this study, we examined which component of mastic gum was responsible for anti-*H. pylori* activity. We prepared the essential oil of mastic gum and identified 20 constituents by GC–MS analysis. Ten standard components were assayed for anti-*H. pylori* activity, and it clarified that *α*-terpineol and (*E*)-methyl isoeugenol showed the anti-*H. pylori* activity against four different *H. pylori* strains that were established from patients with gastritis, gastric ulcer and gastric cancer. These components could be useful to overcome the drug-resistance *H. pylori* growth in stomach.

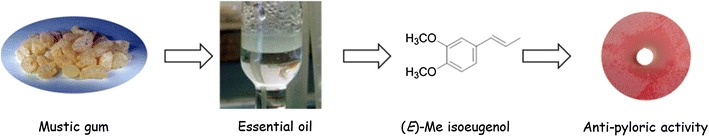

## Introduction

*Helicobacter pylori* (*H. pylori*), gram-negative bacterium, induces chronic gastric infection of one-half of the world population. Infection with *H. pylori* is often associated with a viable proportion of duodenal ulcer, gastric ulcer, and gastric carcinoma [1, 2]. During chronic infection process of *H. pylori* for decades, the bacterium infection initially induces chronic gastritis, and progresses to atrophic gastritis and metaplasia and then to cancer [3]. Intestinal-type gastric cancer shows a multi-step carcinogenic process, from atrophic gastritis to intestinal metaplasia to dysplasia. In Japan, Fukase et al. [4] reported that gastric cancer was inhibited by *H. pylori* eradication in post-endoscopic gastric mucosal resection of gastric cancer in a multi-center randomized control trial. As an approach for prevention of gastric cancer, *H. pylori* infection has been eradicated by combined treatment with several antimiciobial agents plus proton pump inhibitors. However, this approach does not always provide satisfactory benefits for whole population because of the high cost of therapeutic agents and the emergence of antibiotic resistance [5]. A new approach for anti-*H. pylori* vaccines has seen recently developed, and some of these vaccines have had some success in the eradicative animal models, and efforts to prove these vaccines efficaciens are underway in human vaccine trials [6].

Further development of potent antimicrobial drugs is expected to improve the therapeutic and preventive effects against pathogenesis by *H. pylori.* Zaidi et al. [7] have isolated bactericidal constituents from the plant named *Mallotus philippinenesis* that are effective against clarithromycin-and metronidazole-resistant strains of Japanese and Pakistani *H. pylori*. On the other hand, mastic gum, which is a resin secreted from the stem of *Pistacialentiscus,* was found to be effective against *H. pylori* [8]. In our present study, we examined which component of the essential oil prepared from mastic gum could inhibit the growth of *H. pylori.* The bactericidal activity of the mastic components will be discussed in association with their drug resistance reversal effects.

## Results and Discussion

### Chemical Composition of the Essential Oil of Mastic Gum

**Fig. 1:**
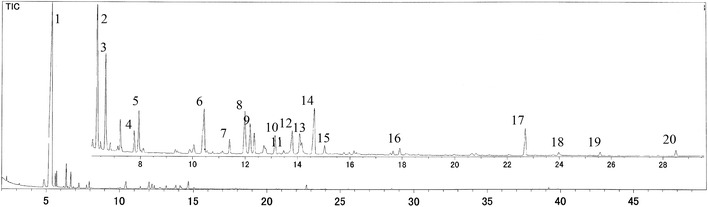
GC–MS chromatogram of the essential oil of mastic gum

GC–MS analysis of the essential oil of mastic gum led to the identification of the components, which are listed in Table 1. A typical GC–MS chromatogram of the essential oil of mastic gum is illustrated in Fig. 1. The identification of the components was based on comparison of their mass spectra with those of NIST and Wiley libraries, as well as on comparison of their retention times [9, 10] and of the standard components analyzed. The major constituent of the essential oil was *α*-pinene (Peak 1; 82.26 %), and totally 20 components were identified from the essential oil of mastic gum.

**Table 1:** Chemical composition of the essential oil of mastic gum

No.	Compound	*t*_R_ (min.)	%	No.	Compound	*t*_R_ (min.)	%
1	*α*-pinene	5.4	82.26	11	*α*-terpineol	13.8	0.77
2	*β*-pinene	6.4	2.96	12	*p*-cymene-8-ol	14.1	0.54
3	*β*-myrcene	6.7	1.92	13	myrtenal	14.2	0.29
4	*p*-cymene	7.8	0.41	14	verbenone	14.7	1.50
5	limonene	7.9	0.84	15	(*E*)-carveol	15.0	0.23
6	linalool	10.4	1.50	16	2-undecanone	17.9	0.16
7	camphenal	11.4	0.31	17	*β*-caryophyllene	22.7	0.73
8	pinocarvenal	12.0	1.25	18	*α*-caryophyllene	24.0	0.09
9	verbenol	12.2	0.71	19	(*E*)-Me isoeugenol	25.6	0.07
10	myrcenol	13.2	0.43	20	caryphyllene oxide	28.5	0.14

### Antibacterial Activity of Mastic Components

**Fig. 2:**
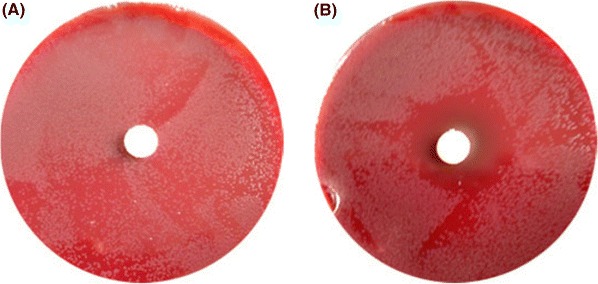
Antibacterial activity of (*E*)-methyl isoeugenol and *α*-terpineol against #09-292 *H. pylori* clinical strain. **a** control, **b** 100 μg/disk of (*E*)-methyl isoeugenol

We next examined which component inhibits the growth of *H. pylori*. Commercially available 10 compounds were tested for antibacterial activities against *H. pylori* clinical strains. The results were summarized in Table 2. Surprisingly, some of them showed antibacterial activity againt clarithromycin (CAM)- and/or metronidazole (MNZ)-resistant strains. Figure 2 shows most potent anti-pyloritic activity of (*E*)-methyl isoeugenol and *α*-terpineol not only against drug sensitive strains (#09-292) but also against drug resistant strains (#09-87, #09-224 and #09-243). These 10 compounds also showed antibacterial activity against three different strains (*E. coli, S. aureus, B. subtilis*) [9].

**Table 2:** Antibacterial activity (zones of inhibition, mm) of the selected compounds of the essential oil of mastic gum

Compounds	μg/disk^a^	#09-87^b^	#09-224^b^	#09-243^b^	#09-292^b^
*α*-pinene	10	–	–	–	–
	100	10	12	–	13
*β*-pinene	10	–	–	–	–
	100	–	–	–	–
*β*-myrcene	10	–	–	10	–
	100	–	–	–	–
*p*-cymene	10	–	–	–	–
	100	11	11	–	–
limonene	10	–	–	–	–
	100	11	11	11	12
linalool	10	–	–	–	–
	100	15	14	14	14
*α*-terpineol	10	–	–	–	–
	100	17	20	18	22
verbenone	10	–	–	–	–
	100	11	11	–	11
*β*-caryophyllene	10	–	–	–	–
	100	12	10	–	–
(*E*)-methyl isoeugenol	10	–	10	–	15
	100	25	23	35	35
essential oil of mastic gum	10	–	–	–	–
	100	–	11	10	–

^a^Antibacterial activity has not been observed at the dose of 1 μg/disk, and data were deleted

^b^#09-87: CAM-sensitive, MNZ-resistant strain; #09-224: CAM-resistant, MNZ-sensitive strain; #09-243: CAM-resistant, MNZ-resistant strain; #09-292: CAM-sensitive, MNZ-sensitive strain

The antibacterial activity of the mastic gum was first reported about two decades ago [11]. In 2007, Paraschos et al. reported that the acidic fraction of mastic gum showed the potent bactericidal activity against *H. pylori* clinical strains and the most active compound was isomasticadienolic acid [12]. In our screening analysis of the essential oil of mastic gum, we identified 20 chemical compositions, and an (*E*)-methyl isoeugenol was found to show the potent antibacterial activity against four *H. pylori* strains including CAM- and MNZ-resistant strain. Another compound, *α*-terpineol also showed antibacterial activity, but its effect was less than that of (*E*)-methyl isoeugenol. In the previous report [9], verbenone, *α*-terpineol, and linalool showed higher antibacterial activity than all other components, and (*E*)-methyl isoeugenol showed moderate activity against *E. coli*, *S. aureus*, *B. subtillis*. (*E*)-methyl isoeugenol and *α*-terpineol are the trace components of the essential oil. However, in our present study, these components first showed much higher antibacterial activity against *H. pylori* when compared with the same dose of the essential oil itself.

*H. pylori* infection is known to be involved in gastric and duodenum ulcer, gastritis and metaplasia, gastric cancer and MALT lymphoma [13–17]. *H. pylori* infection can be eradicated by combined treatment with several antimicrobial agents plus proton pump inhibitors. Fukase et al. have reported that the eradication of *H. pylori* reduces the incidence of gastric cancer [4]. In Japan, combined treatment with proton pump inhibitor (PPI), amoxicillin (AMPC) and CAM have been applied as first line eradication therapy, and MNZ is replaced CAM as second line eradication therapy [18]. However, the subsequent increase in bacterial resistance to CAM in Japan caused a decline in the eradication rate of first line therapy [19]. Our present study demonstrated antibacterial effects of (*E*)-methyl isoeugenol and *α*-terpineol against CAM- and MNZ-resistant *H. pylori* strains (Table 2). These compounds derived from the mastic gum could be further useful for eradication of *H. pylori* including drug-resistant *H. pyroli.*

## Experimental Section

### Material and Chemicals

Mastic gum was purchased from Sunsho Pharmaceutical Co. Ltd. (Fujinomiya, Japan). Standard compounds, *α*-pinene, *β*-pinene, *β*-caryophyllene, terpineol were purchased from Sigma Aldrich Japan (Tokyo, Japan), *β*-myrcene, *p*-cymene, linallol, anethole, verbenone from Wako Pure Chemical Industries, Ltd. (Osaka, Japan), limonene and (*E*)-methyl isoeugenol from Tokyo Chemical Industry Co. Ltd. (Tokyo, Japan).

### Preparation of the Essential Oil of Mastic Gum

The essential oil was prepared according to the manufactural protocol in the Japanese Pharmacopoeia. Mastic gums (30 g) in a 300 mL round bottom flask was set to a prescribed distillation apparatus and refluxing with 300 mL of distilled water for 5 h to yield 0.9 mL of essential oil (731.2 mg, 2.4 % yield).

### Chemical Composition of the Essential Oil of Mastic Gum

The GC–MS analysis of the essential oil was undertaken using a Shimadzu QP-5050A GC–MS system (Kyoto, Japan), operating in electron ionization (EI) mode with an ionization energy of 70 eV. The instrument was equipped with an INERTCAP-5MS/SIL capillary column (30 m, i.d. 0.25 mm, GL Sciences, Tokyo Japan) with helium as carrier gas at 1.4 mL/min flow rate. Column temperature was initially kept for 2 min. at 60 °C, gradually increased to 180 °C at a rate of 3.5 °C/min then increased to 280 °C at a rate of 10 °C/min and kept for 5 min. The injector and interface were set to 300 and 280 °C, respectively, The gas chromatograph operated in the split mode with a split ratio of 94:1. The mass spectrum was monitored starting at *m/z* 40 and ending at *m/z* 500, with a scan interval of 0.5 s. and threshold of 1000, and the solvent cut was set to 2 min. The injection volume was 1 μL. The injection solution was essential oil in acetone (50 % v/v). The chemical composition of the essential oil was analyzed using NIST and Wiley registry of mass spectral data (Shimadzu Corporation).

### *H. pylori* Clinical Strains

In this study, we used four clinical isolates of *H. pylori* which were obtained from patients who underwent endoscopic examination at Oita University Hospital, Oita, Japan. Based on the Epsilometer test (E test) for drug susceptibility, these strains were assessed as sensitive or resistant to clarithromycin (CAM) and metronidazole (MNZ) at minimum inhibitory concentration (MIC) of 1 µg/mL (CAM) and 16 µg/mL or higher (MNZ), respectively. The clinical background and drug sensitivity of the 4 strains were as followed. CAM-sensitive, MNZ-resistant strain #09-87 derived from atrophic gastritis; CAM-resistant, MNZ-sensitive strain #09-224 from gastric ulcer; CAM-resistant, MNZ-resistant strain #09-243 from atrophic gastritis; CAM-sensitive, MNZ-sensitive strain #09-292 from gastric cancer.

### Antibacterial Activity Test

*H. pylori* culture suspension was used to inoculate the plates, and discs containing 1, 10, 100 μg of compounds were applied onto culture plates. The plates were incubated under microaerophilic conditions for 3 days at 37 °C on Mueller Hinton II Agar (Becton Dickinson, Franklin Lakes, NJ, USA) plate supplemented with 7 % horse blood without antibiotics. The antibacterial activities were evaluated by measuring a diameter of the inhibition ring.
